# *Lactobacillus* Strains Alleviated Hyperlipidemia and Liver Steatosis in Aging Rats via Activation of AMPK

**DOI:** 10.3390/ijms21165872

**Published:** 2020-08-16

**Authors:** Lee-Ching Lew, Yan-Yan Hor, Mohamad-Hafis Jaafar, Amy-Sie-Yik Lau, Boon-Kiat Lee, Li-Oon Chuah, Kien-Pong Yap, Azali Azlan, Ghows Azzam, Sy-Bing Choi, Min-Tze Liong

**Affiliations:** 1 School of Industrial Technology, Universiti Sains Malaysia, Penang 11800, Malaysia; leechinglew719@gmail.com (L.-C.L.); yyhor877@gmail.com (Y.-Y.H.); jaafarhafis2733@gmail.com (M.-H.J.); sieyikamy226@gmail.com (A.-S.-Y.L.); boonkiatlee92@gmail.com (B.-K.L.); lioonchuah88@gmail.com (L.-O.C.); 2 USM-RIKEN International Centre for Ageing Science (URICAS), Universiti Sains Malaysia, Penang 11800, Malaysia; azzamghows85@gmail.com; 3 Institute of Biological Sciences, Faculty of Science, University of Malaya, Kuala Lumpur 50603, Malaysia; kianpengyap88@gmail.com; 4 School of Biological Science, Universiti Sains Malaysia, Penang 11800, Malaysia; azaliazlan89@gmail.com; 5 School of Data Sciences, Perdana University, MARDI Complex, Selangor 43400, Malaysia

**Keywords:** triglyceride, immune, *AMPK*, *Lactobacillus*, NAFLD, *SCD1*

## Abstract

In this study, we hypothesized that different strains of *Lactobacillus* can alleviate hyperlipidemia and liver steatosis via activation of 5′ adenosine monophosphate-activated protein kinase (*AMPK*), an enzyme that is involved in cellular energy homeostasis, in aged rats. Male rats were fed with a high-fat diet (HFD) and injected with D-galactose daily over 12 weeks to induce aging. Treatments included (*n* = 6) (i) normal diet (ND), (ii) HFD, (iii) HFD-statin (lovastatin 2 mg/kg/day), (iv) HFD-*Lactobacillus fermentum* DR9 (10 log CFU/day), (v) HFD-*Lactobacillus plantarum* DR7 (10 log CFU/day), and (vi) HFD-*Lactobacillus reuteri* 8513d (10 log CFU/day). Rats administered with statin, DR9, and 8513d reduced serum total cholesterol levels after eight weeks (*p* < 0.05), while the administration of DR7 reduced serum triglycerides level after 12 weeks (*p* < 0.05) as compared to the HFD control. A more prominent effect was observed from the administration of DR7, where positive effects were observed, ranging from hepatic gene expressions to liver histology as compared to the control (*p* < 0.05); downregulation of hepatic lipid synthesis and β-oxidation gene stearoyl-CoA desaturase 1 (*SCD1*), upregulation of hepatic sterol excretion genes of ATP-binding cassette subfamily G member 5 and 8 (*ABCG5* and *ABCG8*), lesser degree of liver steatosis, and upregulation of hepatic energy metabolisms genes *AMPKα1* and *AMPKα2*. Taken altogether, this study illustrated that the administration of selected *Lactobacillus* strains led to improved lipid profiles via activation of energy and lipid metabolisms, suggesting the potentials of *Lactobacillus* as a promising natural intervention for alleviation of cardiovascular and liver diseases.

## 1. Introduction

Aging is defined as a time-dependent progressive deteriorative change that increases vulnerability to challenges and decreases the ability to survive [1]. Aging is often associated with metabolic diseases, such as hyperlipidemia, that increase the risk of cardiovascular diseases (CVD). According to the World Health Organization, on a global level, CVD accounts for 31% of all deaths, and cardiovascular complications are considered as the main factors of morbidity and mortality. The estimated cost of CVD will exceed one billion Euros by 2030.

Elevated levels of blood lipid, or hyperlipidemia, are well-documented risk factors for CVD. Hyperlipidemia can broadly be classified as isolated elevation of cholesterol, isolated elevated triglyceride (TG), and elevations of both [2]. Hyperlipidemia has also been shown to affect the antioxidant status of different organs, as well as their lipoprotein levels, which in turn can intensify metabolic disturbances and increases the risk of cardiovascular diseases [3], as well as nonalcoholic fatty liver disease (NAFLD) [4]

The use of statin has resulted in significant improvement in morbidity and mortality from CVD [5]. Statin was shown to reduce CVD via reduction of low-density lipoprotein cholesterol (LDL-C) [6]. However, even with the substantial LDL-C lowering achieved with statin treatments, these drugs have reduced coronary heart disease (CHD)-associated mortality and morbidity by only ~30% [7]. Reports are now showing that cardiac events occur in people even without clinically abnormal LDL-C concentrations, suggesting that LDL-C is not the only parameter that need to be monitored [2]. Suggestion to improve the risk prediction and treatment has been to focus on non-high-density-lipoprotein (HDL) cholesterol (includes LDL and other intermediate or end products of triglyceride-rich lipoprotein) levels rather than on just LDL-C [8]. Probiotics, such as those from the genus of *Lactobacillus*, have been reported to have a long history of safe use, primarily for gut health, antimicrobial properties [9,10], and now in metabolic diseases including hypertension [11] for improvement of blood lipid profiles and reducing total cholesterol (TC) and TG concentrations in blood [12,13]. A recent systematic review and a meta-analysis of 15 randomized controlled trials reported that probiotics consumption was able to decrease total-cholesterol and LDL-cholesterol levels effectively [14].

Conventionally, *AMPK* has been viewed as a main control governing systemic and cellular energy status while protecting cellular function under energy-restricted conditions. Recent evidence has shown that *AMPK* can be activated by varying responses, ranging from metabolic stresses, glucose deprivation, exercise, and muscle contraction. Activation of *AMPK* initiates the reprogramming of metabolism, leading to a restored energy balance, making *AMPK* a key regulator of crucial biological pathways. We have recently reported the ability of Lactobacillus strains to activate the *AMPK* pathway in mammalian cell cultures via phosphorylation [15]. In addition to controlling energy metabolism, *AMPK* has also been shown as a key player in the regulation of hepatic lipid metabolism via lowering hepatic TG level and oxidation of hepatic fat [16].

It is worth noting that the effects, mechanisms, and targets of probiotics are strain-specific. In this study, we aimed to study the effects of *Lactobacillus fermentum DR9*, *Lactobacillus plantarum* DR7, and *Lactobacillus reuteri* 8513d on the lipid metabolism in an aging rat model. While past studies have used a high-fat diet to induce aging, recent studies have shown that aging can also be accelerated via chronic administration of D-galactose, primarily via accumulation of reactive oxygen species, as well as through glycation of end products [17]. The D-galactose-induced model has also been reported to exhibit conditions similar to naturally aged animals [18]. Considering that we have found *AMPK* activation potentials of certain *Lactobacillus* strains, we hypothesized that some of these strains can also regulate lipid metabolism, alleviate hyperlipidemia, and restore liver conditions, such as that of steatosis. We postulate that this study will advance the field of nutrition through our efforts to use probiotics a natural dietary intervention against cardiovascular and liver diseases.

## 2. Results

### 2.1. Body Weight, Feed Intake, Feed Efficacy, Fasting Blood Glucose, Adipose Weight, and Percent Body Fat

**Figure 1:**
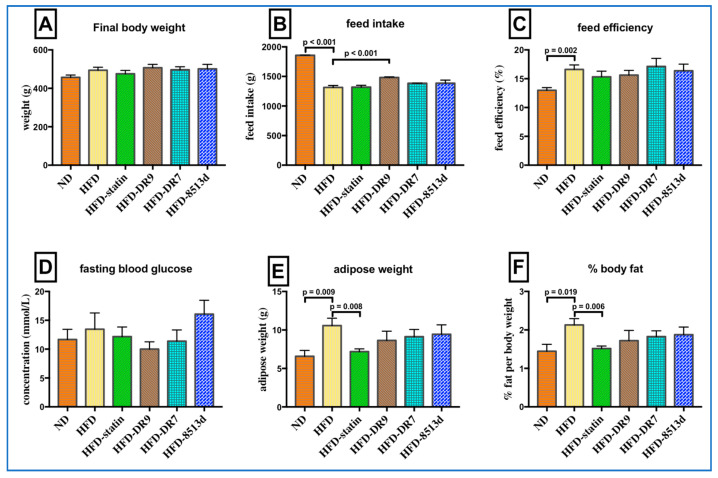
Final body weight (**A**), feed intake (**B**), feed efficiency (**C**), fasting blood glucose (**D**), adipose weight (**E**), and percent body fat (**F**) in D-galactose-induced aging rats (600 mg/kg/day) after 12 weeks of treatment. ND (normal diet), HFD (high-fat diet), HFD-statin (high-fat diet, receiving lovastatin 2 mg/kg/day), HFD-DR9 (high-fat diet, receiving *L. fermentum* DR9 (10^10^ CFU/day), HFD-DR7 (high-fat diet, *receiving L. plantarum* DR7 (10^10^ CFU/day), HFD-8513d (high-fat diet, receiving *L. reuteri* 8513d (10^10^ CFU/day). Results are expressed as mean; error bars (SEM); *n* = 6. Results are expressed as mean; error bars (SEM); *n* = 6. Statistical analysis was performed using independent *t*-tests. * Food efficiency, weight gain (g)/feed intake (g) × 100.

The final body weight, food intake and efficiency, fasting blood glucose, adipose weight, and percentage of body fat of the rats after 12 weeks of treatment are as shown in Figure 1. There was no significant difference in final body and fasting blood glucose when comparing between all the treatment groups. Feed intake was highest in the ND group. The HFD-DR9 group showed a significantly higher (*p* < 0.05) feed intake compared to the HFD group.

Feed efficiency was significantly higher (*p* < 0.05) in the HFD group as compared to the ND group. It was also observed that the HFD group had a significant higher (*p* < 0.05) adipose weight and percent body fat as compared to the ND group. Results also showed that the HFD-statin group had a significantly lower (*p* < 0.05) adipose weight and percent body fat as compared to the HFD group. There was no significant difference (*p* > 0.05) in adipose weight and percent body fat when comparing the HFD-DR9, HFD-DR7, and HFD-8513d groups with the HFD group.

### 2.2. Serum Biochemical Analysis

#### 2.2.1. Serum Lipid Profile

**Figure 2:**
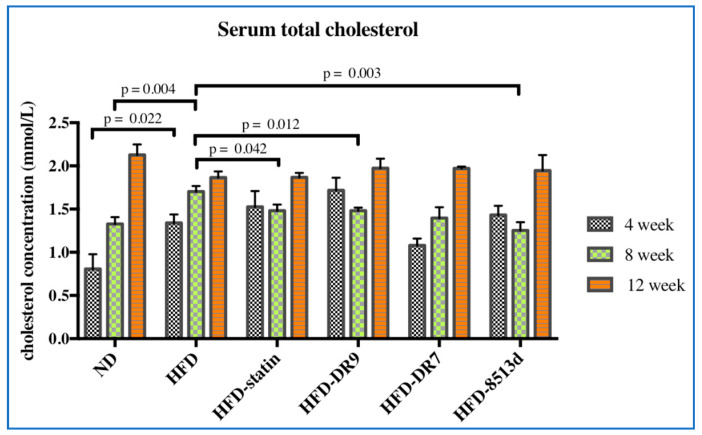
Serum total cholesterol in D-galactose-induced aging rats (600 mg/kg/day) after 4, 8, and 12 weeks of treatment. ND (normal diet), HFD (high-fat diet), HFD-statin (high-fat diet, receiving lovastatin 2 mg/kg/day), HFD-DR9 (high-fat diet, receiving *L. fermentum* DR9 (10^10^ CFU/day), HFD-DR7 (high-fat diet, *receiving L. plantarum* DR7 (10^10^ CFU/day), HFD-8513d (high-fat diet, receiving *L. reuteri* 8513d (10^10^ CFU/day). Results are expressed as mean; error bars (SEM); *n* = 6. Statistical analysis was performed using independent *t*-tests.

Serum total cholesterol levels were as shown in Figure 2. After four weeks of treatment, it was observed that the HFD group had a significantly higher (*p* < 0.05) total cholesterol level compared to the ND group. No significant changes in the total cholesterol level were observed between the HFD group and the HFD-statin, HFD-DR9, HFD-DR7, and HFD-8513d groups after four weeks of treatment. However, after eight weeks of treatment, results showed that the HFD-statin, HFD-DR9, and HFD-8513d groups had significantly lower (*p* < 0.05) serum total cholesterol level compared to the HFD group. No significant difference between the different treatment groups was observed at week 12.

**Figure 3:**
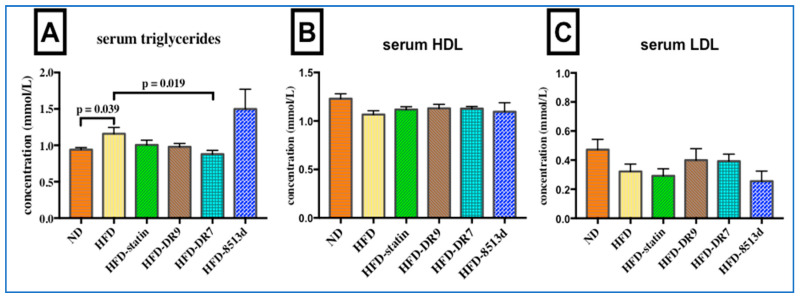
Serum triglyceride (**A**), HDL (**B**), and LDL (**C**) in D-galactose-induced aging rats (600 mg/kg/day) after 12 weeks of treatment. ND (normal diet), HFD (high-fat diet), HFD-statin (high-fat diet, receiving lovastatin 2 mg/kg/day), HFD-DR9 (high-fat diet, receiving *L. fermentum* DR9 (10^10^ CFU/day), HFD-DR7 (high-fat diet, *receiving L. plantarum* DR7 (10^10^ CFU/day), HFD-8513d (high-fat diet, receiving *L. reuteri* 8513d (10^10^ CFU/day). Results are expressed as mean; error bars (SEM); *n* = 6. Statistical analysis was performed using independent *t*-tests.

Serum triglyceride, HDL, and LDL levels in rats after 12 weeks of treatments were as shown in Figure 3. Results showed that serum triglyceride level was significantly increased (*p* < 0.05) when the rats were fed with high-fat diet. Only the HFD-DR7 group showed a significantly lower (*p* < 0.05) triglyceride level when compared to the HFD group. No significant changes in serum HDL and LDL level was observed when comparing between the different groups of rats after 12 weeks of treatment.

#### 2.2.2. Liver Function Test

**Figure 4:**
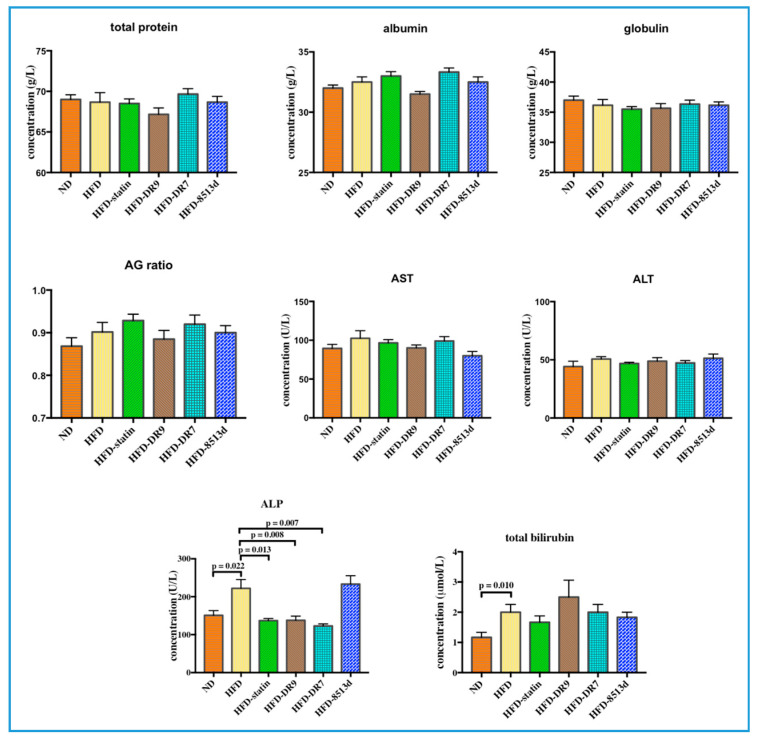
Liver function test, including total protein, albumin, globulin, AG ratio (Albumin/Globulin ratio), AST (aspartate aminotransferase), ALT (alanine aminotransferase), ALP (alkaline phosphatase), and total bilirubin in D-galactose-induced aging rats (600 mg/kg/day) after 12 weeks of treatment. ND (normal diet), HFD (high-fat diet), HFD-statin (high-fat diet, receiving lovastatin 2 mg/kg/day), HFD-DR9 (high-fat diet, receiving *L. fermentum* DR9 (10^10^ CFU/day), HFD-DR7 (high-fat diet, *receiving L. plantarum* DR7 (10^10^ CFU/day), HFD-8513d (high-fat diet, receiving *L. reuteri* 8513d (10^10^ CFU/day). Results are expressed as mean; error bars (SEM); *n* = 6. Statistical analysis was performed using independent *t*-tests.

The liver function test results, which include total protein, albumin, globulin, AG ratio (Albumin/Globulin ratio), AST (aspartate aminotransferase), ALT (alanine aminotransferase), ALP (alkaline phosphatase), and total bilirubin, are shown in Figure 4. Total bilirubin and ALP were significantly increased (*p* < 0.05) in the HFD group compared to the ND group. Administration of statin, *L. fermentum* DR9, and *L. plantarum* DR7 to rats fed with HFD did not cause any significant changes in all the liver function parameters except for the ALP test, where the concentration was significantly reduced (*p* < 0.05) when compared to the HFD group. The HFD-8513d group did not show any significant difference on all the liver function parameters when compared to the HFD group.

#### 2.2.3. Renal Function Test

**Figure 5:**
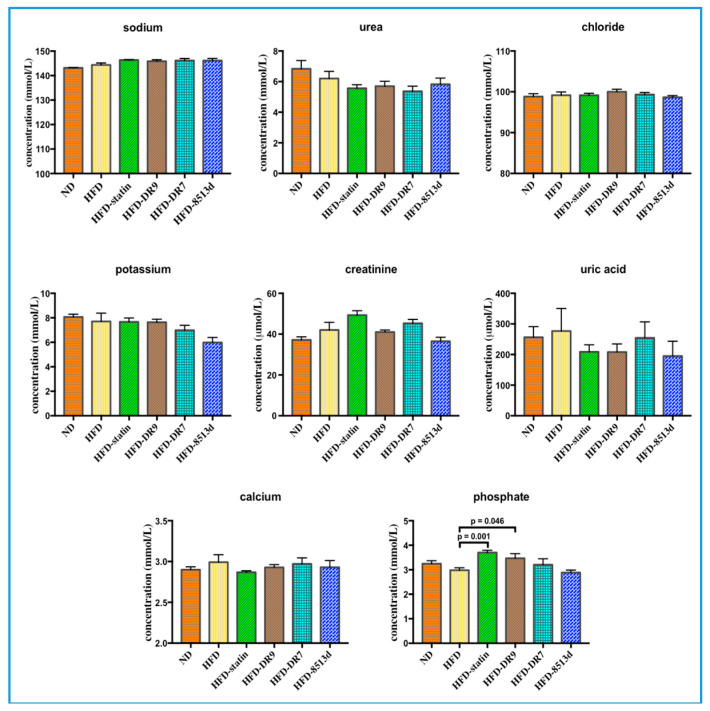
Renal function test, including sodium, urea, chloride, potassium, creatinine, uric acid, calcium, and phosphate of D-galactose-induced aging rats (600 mg/kg/day) after 12 weeks of treatment. ND (normal diet), HFD (high-fat diet), HFD-statin (high-fat diet, receiving lovastatin 2 mg/kg/day), HFD-DR9 (high-fat diet, receiving *L. fermentum* DR9 (10^10^ CFU/day), HFD-DR7 (high-fat diet, *receiving L. plantarum* DR7 (10^10^ CFU/day), HFD-8513d (high-fat diet, receiving *L. reuteri* 8513d (10^10^ CFU/day). Results are expressed as mean; error bars (SEM); *n* = 6. Statistical analysis was performed using independent *t*-tests.

The renal function test results, which include sodium, urea, chloride, potassium, creatinine, uric acid, calcium, and phosphate, are shown in Figure 5. Results from this study showed that administration of *L. fermentum* DR9, *L. plantarum* DR7, and *L. reuteri* 8513d to rats fed with HFD did not cause any significant changes in levels of sodium, urea, chloride, potassium, creatinine, uric acid, and calcium. Serum phosphate was significantly higher (*p* < 0.05) in the HFD-statin and HFD-DR9 groups compared to the HFD group.

### 2.3. Gene Expression of Lipid Metabolism Pathway

#### 2.3.1. Stearoyl-CoA Desaturase 1 (*SCD1*)

**Figure 6:**
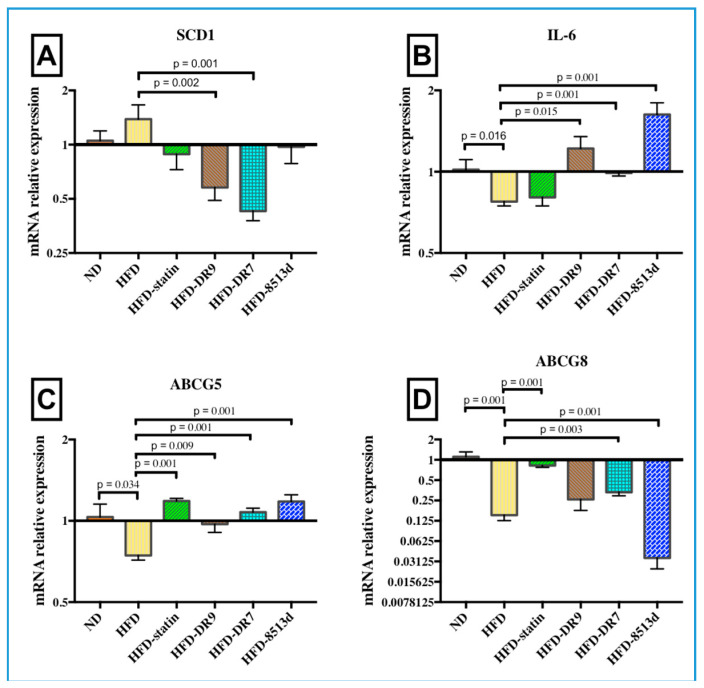
Relative gene expression of (**A**) *SCD1*, (**B**) *IL-6*, (**C**) *ABCG5*, and (**D**) *ABCG8* in liver of D-galactose-induced aging rats (600 mg/kg/day) after 12 weeks of treatment. ND (normal diet), HFD (high-fat diet), HFD-statin (high-fat diet, receiving lovastatin 2 mg/kg/day), HFD-DR9 (high-fat diet, receiving *L. fermentum* DR9 (10^10^ CFU/day), HFD-DR7 (high-fat diet, receiving *L. plantarum* DR7 (10^10^ CFU/day), HFD-8513d (high-fat diet, receiving *L. reuteri* 8513d (10^10^ CFU/day). Results are expressed as mean; error bars (SEM); *n* = 6. Statistical analysis was performed using independent *t*-tests.

*SCD1* is a delta-9 fatty acid desaturase that catalyzes the synthesis of mono-unsaturated fatty acids (MUFA) and is a critical control point regulating hepatic lipid synthesis and β-oxidation. Results from quantitative real-time PCR analysis showed that mRNA expression levels of *SCD1* in liver were significantly downregulated (*p* < 0.05) in the HFD-DR9 and HFD-DR7 groups (Figure 6A) when compared to the HFD group.

#### 2.3.2. Interleukin-6 (*IL-6*)

The relative gene expression of *IL-6* in liver after 12 weeks of treatment is shown in Figure 6B. Gene expression of *IL-6* was significantly lower (*p* < 0.05) in the HFD group compared to the ND group. It was also observed that administration of *L. fermentum* DR9, *L. plantarum* DR7, and *L. reuteri* 8513d significantly upregulated the expression of *IL-6* (*p* < 0.05).

#### 2.3.3. ATP-Binding Cassette Subfamily G Member 5 and 8 (*ABCG5* and *ABCG8*)

*ABCG5* and *ABCG8* are half-size ABC transporters that function as heterodimers (*ABCG5/G8*) to increase sterol excretion from the liver. The relative gene expression of *ABCG5* and *ABCG8* in liver after 12 weeks of treatment is shown in Figure 6C,D. Results from this study showed that high-fat diet significantly reduced the mRNA expression of *ABCG5* and *G8* (*p* < 0.05). The HFD-statin, HFD-DR9, HFD-DR7, and HFD-8513d groups had significantly higher mRNA expression of *ABCG5* (*p* < 0.05). Expression of *ABCG8* was also significantly upregulated upon administration of statin and *L. plantarum* DR7 (*p* < 0.05).

#### 2.3.4. Scavenger Receptor B1 (*SR-B1*)

**Figure 7:**
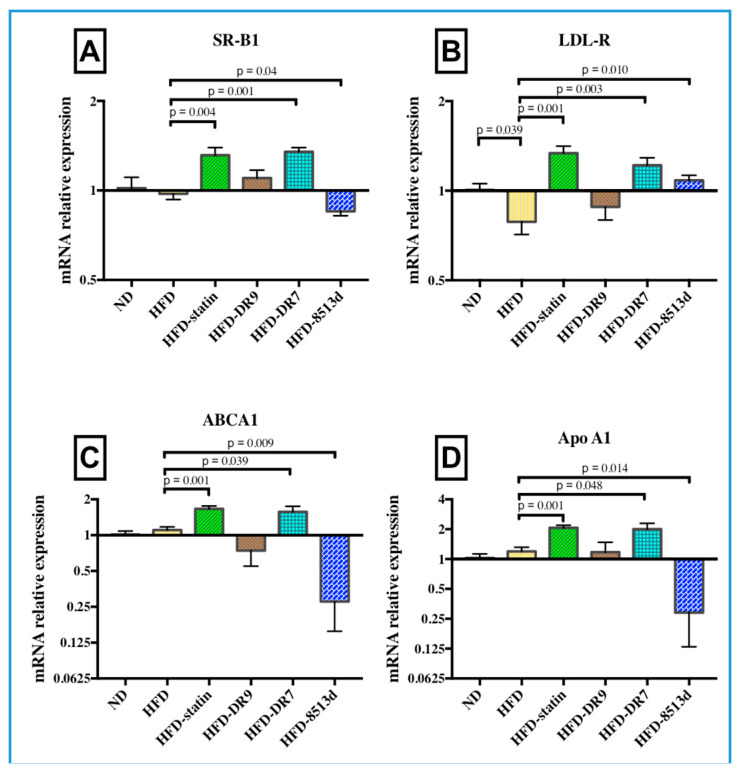
Relative gene expression of (**A**) *SR-B1*, (**B**) *LDL-R*, (**C**) *ABCA1*, and (**D**) *Apo A1* in liver of D-galactose-induced aging rats (600 mg/kg/day) after 12 weeks of treatment. ND (normal diet), HFD (high-fat diet), HFD-statin (high-fat diet, receiving lovastatin 2 mg/kg/day), HFD-DR9 (high-fat diet, receiving *L. fermentum* DR9 (10^10^ CFU/day), HFD-DR7 (high-fat diet, receiving *L. plantarum* DR7 (10^10^ CFU/day), HFD-8513d (high-fat diet, receiving *L. reuteri* 8513d (10^10^ CFU/day). Results are expressed as mean; error bars (SEM); *n* = 6. Statistical analysis was performed using independent *t*-tests.

The relative gene expression of *SR-B1* in liver after 12 weeks of treatment is shown in Figure 7A. It was observed from this study that *SR-B1* expression was significantly lower in the HFD group compared to the ND group (*p* < 0.05). The results also showed that the HFD-statin and HFD-DR7 groups exhibited a significantly higher expression of *SR-B1* (*p* < 0.05) compared to the HFD group. Meanwhile, the HFD-8513d group showed significantly lower *SR-B1* expression (*p* < 0.05) when compared to the HFD group.

#### 2.3.5. Low Density Lipoprotein Receptor (*LDL-R*)

The relative gene expression of *LDL-R* in liver after 12 weeks of treatment is shown in Figure 7B. It was observed that the expression of *LDL-R* was significantly downregulated upon administration of high-fat diet (*p* < 0.05). Results also showed that *LDL-R* mRNA expression was significantly higher in the HFD-statin, HFD-DR7, and HFD-8513d groups when compared to the HFD group (*p* < 0.05).

#### 2.3.6. ATP-Binding Cassette Subfamily A Member 1 (*ABCA1*)

The relative gene expression of *ABCA1* in liver after 12 weeks of treatment is shown in Figure 7C. No significant changes in *ABCA1* mRNA expression were observed when comparing between the ND and HFD groups. However, it was observed that the HFD-statin and HFD-DR7 group had significantly higher (*p* < 0.05) expression of *ABCA1* compared to the HFD group. On the other hand, results showed that the HFD-8513d group had a significantly lower (*p* < 0.05) expression of *ABCA1* when compared to the HFD group.

#### 2.3.7. Apolipoprotein A1 (*Apo A1*)

The relative gene expression of *ApoA1* in liver after 12 weeks of treatment is shown in Figure 7D. Similar trends were observed when comparing between *ApoA1* and *ABCA1* gene expression; the HFD-statin and HFD-DR7 groups had significantly higher (*p* < 0.05) expression of *Apo A1* compared to the HFD group. The HFD-8513d group had a significantly lower (*p* < 0.05) expression of *Apo A1* when compared to the HFD group. In addition, there were no significant changes in *Apo A1* mRNA expression when comparing between the ND and HFD groups.

#### 2.3.8. 5′ Adenosine Monophosphate-Activated Protein Kinase (*AMPK*)

**Figure 8:**
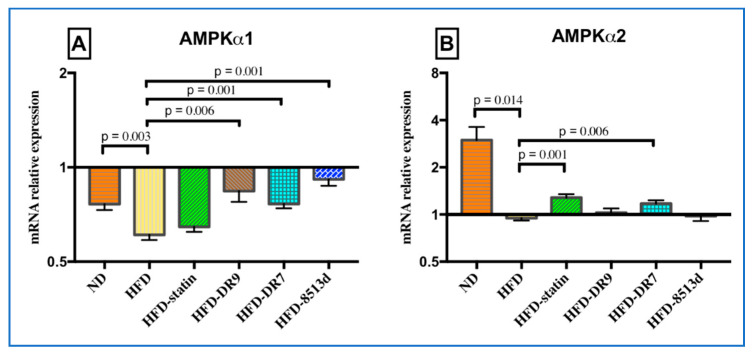
Relative gene expression of (**A**) *AMPKα1* (**B**) *AMPKα2* in liver of D-galactose-induced aging rats (600 mg/kg/day) after 12 weeks of treatment. ND (normal diet), HFD (high-fat diet), HFD-statin (high-fat diet, receiving lovastatin 2 mg/kg/day), HFD-DR9 (high-fat diet, receiving *L. fermentum* DR9 (10^10^ CFU/day), HFD-DR7 (high-fat diet, receiving *L. plantarum* DR7 (10^10^ CFU/day), HFD-8513d (high-fat diet, receiving *L. reuteri* 8513d (10^10^ CFU/day). Results are expressed as mean; error bars (SEM); *n* = 6. Statistical analysis was performed using independent *t*-tests.

The relative gene expression of *AMPKα1* and *AMPKα2* in liver after 12 weeks of treatment is shown in Figure 8A,B, respectively. Results showed that both the mRNA expression of *AMPKα1* and *AMPKα2* were significantly lower in the HFD group as compared to the ND group. The HFD-DR9, HFD-DR7, and HFD-8513d groups showed significantly higher expression of *AMPKα1* compared to the HFD group. Meanwhile, only the HFD-statin and HFD-DR7 groups had significantly higher mRNA expression of *AMPKα2* when compared to the HFD group.

### 2.4. Liver Immune Response: IL-1β, IL-4, IL-10, TNF-α, and IFN-γ

**Figure 9:**
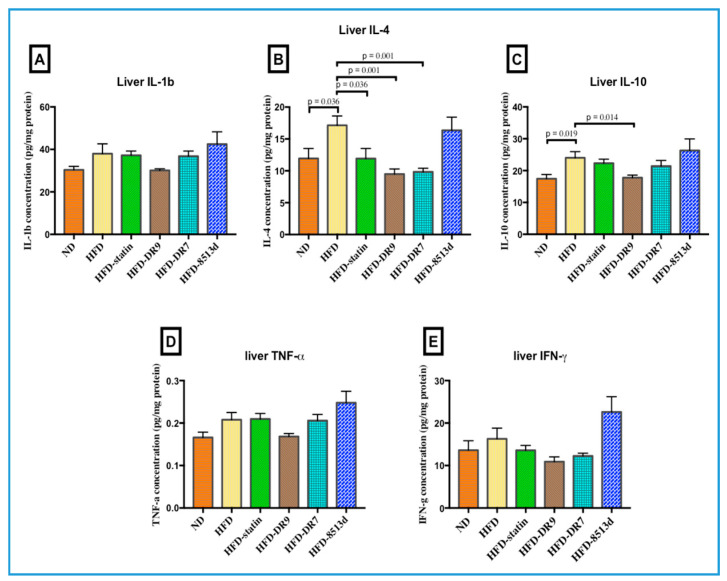
Cytokine levels of (**A**): Interleukin 1-beta (IL-1b), (**B**): Interleukin-4 (IL-4), (**C**): Interleukin-10 (IL-10), (**D**): Tumor necrosis factor-alpha (TNF-α), (**E**): Interferon-gamma (IFN-γ)) in liver of D-galactose-induced aging rats (600 mg/kg/day) after 12 weeks of treatment. ND (normal diet), HFD (high-fat diet), HFD-statin (high-fat diet, receiving lovastatin 2 mg/kg/day), HFD-DR9 (high-fat diet, receiving *L. fermentum* DR9 (10^10^ CFU/day), HFD-DR7 (high-fat diet, receiving *L. plantarum* DR7 (10^10^ CFU/day), HFD-8513d (high-fat diet, receiving *L. reuteri* 8513d (10^10^ CFU/day). Results are expressed as mean; error bars (SEM); *n* = 6. Statistical analysis was performed using independent *t*-tests.

Protein expression (Interleukin-1beta (IL-1β), Interleukin-4 (IL-4), Interleukin-10 (IL-10), Tumor necrosis factor alpha (TNF-α), Interferon gamma (IFN-γ)) in liver tissue after 12 weeks of treatment was measured using Multiplex ELISA and is shown in Figure 9. There was no significant difference in liver IL-1b, TNF-α, and IFN-γ when comparing between the groups. Results showed that IL-4 was significantly increased in the HFD group compared to the ND group. Compared to the HFD group, the HFD-statin, HFD-DR9, and HFD-DR7 groups had lower (*p* < 0.05) IL-4 levels. The liver IL-10 level was significantly higher (*p* < 0.05) in the HFD group as compared to the ND group. The HFD-DR9 group had a lower (*p* < 0.05) level of IL-10 than the HFD group.

### 2.5. Liver Histology

**Figure 10:**
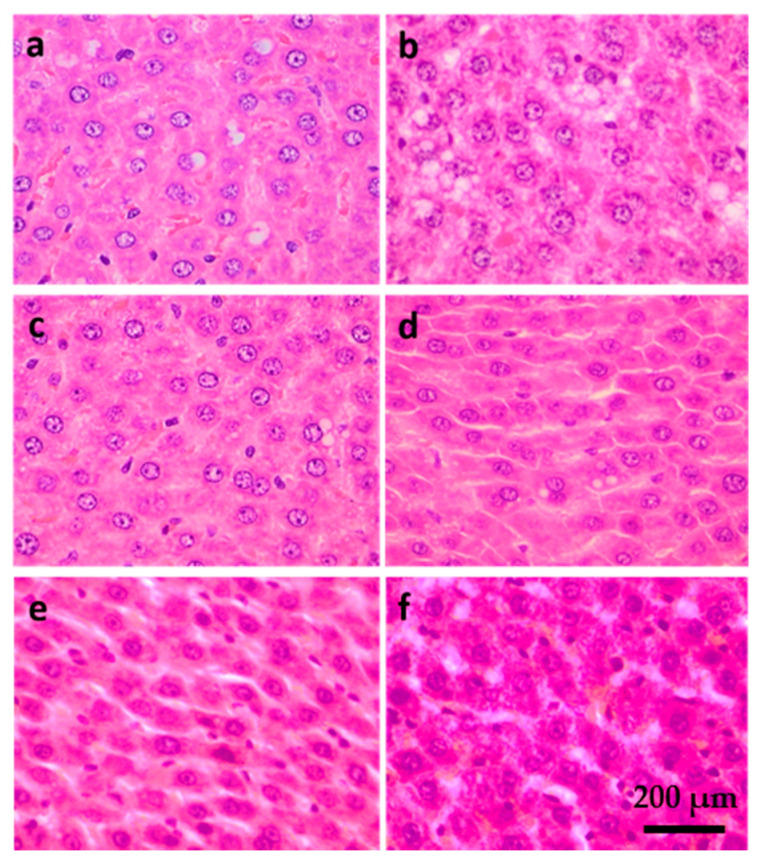
Hematoxylin and eosin (H&E) staining of liver sections of D-galactose-induced aging rats (600 mg/kg/day) after 12 weeks of treatment. (**a**) ND (normal diet), (**b**) HFD (high-fat diet), (**c**) HFD-statin (high-fat diet, receiving lovastatin 2 mg/kg/day), (**d**) HFD-DR7 (high-fat diet, receiving *L. plantarum* DR7 (10^10^ CFU/day), (**e**) HFD-DR9 (high-fat diet, receiving *L. fermentum* DR9 (10^10^ CFU/day), (**f**) HFD-8513d (high-fat diet, receiving *L. reuteri* 8513d (10^10^ CFU/day).

Samples stained with hematoxylin and eosin (H&E) are shown in Figure 10. A higher degree of lipid accumulation and less organized structure were observed in the HFD group when compared to the ND group. The HFD-statin, HFD-DR7, HFD-DR9, and HFD-8513d groups showed less lipid accumulation compared to the HFD group. Interestingly, a more well-organized structure was observed for the HFD-DR7 and HFD-DR9 groups compared to the other treatment groups.

## 3. Discussion

Hyperlipidemia, which refers to increased levels of cholesterol and triglycerides in the blood, is among the known risk factors for the development of cardiovascular diseases. The term hyperlipidemia also includes disorders of lipoprotein metabolism that lead to atherosclerosis. Probiotics have been shown to improve the blood lipid profile both in animal and human clinical studies. Increasing studies have shown the ability of lactobacilli in reducing TC and TG concentrations [13,19,20,21]. Several mechanisms have been proposed; however, it must be noted that the proposed mechanism for one strain should not be extrapolated to another strain. In this study, we evaluated the effect of three probiotic strains on D-galactose-induced aging model rats fed with a high-fat diet. The current study showed that probiotic strains *L. fermentum* DR9 and *L. reuteri* 8513d have a positive effect on serum TC after eight weeks of treatment. However, there was no significant difference between the groups upon continuous feeding of HFD to week 12. This result suggested that probiotics may be useful in delaying the increase of TC, but continuously consuming HFD may mask the cholesterol-lowering effect. Several cholesterol-lowering mechanisms that lead to improvement of the serum lipid profile have been reported. Probiotics have been reported to assimilate cholesterol, thereby lowering luminal cholesterol levels available for absorption [22], as well as via bile salt hydrolase activity [10]. Probiotics have also been reported to combat hypercholesterolemia via alteration of the gut microbiota profile [23].

Interestingly, we observed that although *L. plantarum* DR7 did not significantly affect the serum TC level, serum TG level was significantly reduced after 12 weeks of treatment compared to the control. TG has now been recognized as a strong marker for CVD and diabetes, as well as NAFLD [4]. Probiotics have been reported to exert their lipid-modulating effect by regulating the host genes responsible for lipid metabolism [20,24,25]. In this study, it was observed that *L. plantarum* DR7 and *L. fermentum* DR9 significantly downregulated the gene expression of *SCD1* in the liver, where the former showed a more prominent effect. *SCD1* is a rate-limiting enzyme that converts saturated fatty acids into MUFAs, mainly oleate (18:1) and palmitoleate (16:1), which represent the major MUFAs of membrane phospholipids, TGs, and cholesterol esters [26]. Previous studies have shown that *SCD1*-deficient mice are protected from hypertriglyceridemia largely through increased fatty acid oxidation and reduced fatty acid synthesis in the liver [27]. It was suggested that *SCD1* activity might be rate-limiting for triglyceride production in a wide array of dyslipidemias [28]. We thus postulated that *L. plantarum* DR7 exerted its TG-lowering effect via downregulation of *SCD1* gene expression.

Inhibition of *SCD1* has also been reported to activate *AMPK* using in vitro [29] and in vivo models where global deletion of *SCD1* in mice resulted in activation of *AMPK* in the liver [30] and skeletal muscle [31]. *AMPK* is an energy sensor that plays an important role in sustaining cellular energy levels [32] and thus has emerged as a potential therapeutic target for metabolic diseases. *AMPK* is also the upstream kinase for the critical metabolic enzymes such as HMG-CoA reductase, the rate-limiting enzyme responsible for de novo synthesis of cholesterol in the liver [33]. When activated, *AMPK* phosphorylates downstream pathways that are critical for lipid and carbohydrate metabolism [34]. More studies have now suggested increasing the activity of *AMPK* as a treatment strategy for hyperlipidemia [35,36]. Exercise has also been reported to increase *AMPK* activity [37]. In our previous in vitro study using liver cell line HepG2, we showed that *L. plantarum* DR7 could activate *AMPK* [15]. This observation was further corroborated in our current in vivo study using rats where expression of both *AMPK* subunits, α-1 and α-2, was significantly upregulated in the liver. *AMPK*’s main catalytic subunit alpha has two isoforms, α1 and α2, which are widely expressed in cardiac muscle and liver. All these indicated that *L. plantarum* DR7 might be activating *AMPK* via downregulation of *SCD1* gene expression.

NAFLD is characterized by excessive lipid accumulation in hepatocytes in the form of TG, and elevated serum TG has now been proven to be a strong marker for NAFLD in a cohort study [4]. Histology analysis showed that the HFD group liver contained more lipid droplets in the form of macrovesicular and microvesicular steatosis. Administration of *L. plantarum* DR7, *L. fermentum* DR9 and statin reduced the accumulation of lipid in the liver and showed lesser degree of steatosis as compared to the HFD group. We hypothesized that the TG-lowering effects of probiotics may lead to alleviation of NAFLD symptoms in rats fed with HFD. Reduction in serum ALP was also observed in the *L. plantarum* DR7, *L. fermentum* DR9, and statin-treated groups. Elevated ALP has also been shown to be a risk factor for nonalcoholic fatty liver disease [38]. It has been reported that consumption of high-fat meal led to higher serum ALP [39]. ALP in the blood is primarily derived from excess ALP that is released from cells of the liver, bones, and bile ducts. High levels of ALP may indicate liver and bone diseases. A rise in ALP activity occurs with all forms of cholestasis (decrease in bile flow due to impaired secretion or obstruction of bile flow). In a recent large prospective study involving 132,377 adults, a strong correlation between ALP concentration and metabolic disorders, such as type-2 diabetes, was observed [40]. Potential of probiotics in regulation of serum ALP has been shown previously in an animal study using rats where administration of *L. plantarum* and *L. casei* significantly reduced the serum ALP concentration [41].

In this study, it was also observed that probiotic strains *L. plantarum* DR7, *L. fermentum* DR9, and *L. reuteri* 8513d significantly increased the expression of *IL-6* in the liver tissue. Although *IL-6* has been reported to exhibit both pro- and anti-inflammatory properties, there is also information indicating that this cytokine can protect against cardiovascular diseases [42]. Interleukin-6 has been reported to exhibit atheroprotective effects by lowering of plasma LDL via upregulation of LDL receptor gene expression [43]. *LDL-R* expression was higher in *L. plantarum* DR7, *L. reuteri* 8513d, and statin-treated groups. *IL-6* has also been reported to exert a positive impact on lipid metabolism through upregulation of *ABCA1* by increasing the cholesterol efflux to *Apo A1* in macrophages [44], and thus reduces the risk of CVD. *ABCA1* is the rate-controlling protein in the *Apo A1*-dependent active transport of cholesterol and phospholipids [45]. SR-B1, on the other hand, promotes cholesterol efflux from cells to lipid-poor *Apo A1* [46]. Cholesterol from peripheral tissue, carried by HDL, is metabolized in the liver after uptake by the *SR-B1* [47]. In this study, it was observed that *ABCA1*, *Apo A1*, and *SR-B1* expression was increased in the HFD-DR7 and HFD-statin group. This study also showed that *ABCG5* is upregulated upon treatment with *L. plantarum* DR7, *L. fermentum* DR9, *L. reuteri* 8513d and statin. However, *ABCG8* is only upregulated upon treatment with *L. plantarum* DR7. *ABCG5* and *G8* have been reported to play a critical role in maintaining sterol balance via a reverse cholesterol transport mechanism. It has been reported that increased expression of *ABCG5* and *ABCG8* selectively drives biliary neutral sterol secretion and reduces intestinal cholesterol absorption. The ability of probiotic strains to modulate the expression of *ABCG5* and *G8* has also been reported from several previous in vitro studies using cell lines [48,49].

Experimental data from this study also showed that HFD increased liver IL-4 level. It was also observed that liver IL-4 levels decreased in the HFD-DR9, HFD-DR7, and HFD-statin groups. According to a recent review, IL-4 is a complicated cytokine whose role varies between anti- and proinflammation in autoimmunity [50]. However, in experimental, immune-mediated liver injury, IL-4 is clearly proinflammatory, where the target cells can be CD4+ T cells or macrophages [48]. Of note, the level of anti-inflammatory cytokine IL-10 was persistently elevated in the HFD group, and administration of *L. fermentum* DR9 decreased the IL-10 level. A similar observation was reported in previous studies in mice [51,52] and humans [53] where HFD increased hepatic IL-10 levels, and likely represents a protective response of the body to counteract proinflammatory events.

In conclusion, it was observed that administration of different probiotic strains may lead to alteration of lipid metabolism in different ways, as shown in their different gene and protein expression profiles. We hypothesized that probiotic strains with potential in reducing TG will be a promising natural intervention for alleviation of CVD as well as NAFLD.

## 4. Materials and Methods

### 4.1. Bacterial Culture

*Lactobacillus reuteri* 8513d was obtained from School of Industrial Technology, Universiti Sains Malaysia (Penang, Malaysia). *Lactobacillus plantarum* DR7 and *Lactobacillus fermentum* DR9 were isolated from fresh cow’s milk from Penang and were obtained via courtesy of Clinical Nutrition Intl (M) Sdn. Bhd., Malaysia. The screening and selection of these strains are as previously described [15]. All stock cultures were preserved in 20% (*v*/*v*) glycerol (−20 °C), activated in sterile de Mann, Rogosa, Sharpe (MRS) broth (Hi-media, Mumbai, India) for three successive times using 10% (*v*/*v*) inoculums and incubated at 37 °C for 24 h prior to use.

### 4.2. Animal Experiments

All animal experiments were approved by the USM Animal Care and Use Committee (USM/Animal Ethics Approval/2016/(724)) and were carried out under the institutional guidelines for ethical animal use. Sprague–Dawley rats at eight weeks of age were used in this study. Animals were housed with alternating 12 h light and dark cycles with free access to food and water. Food intake and body weight were recorded once a week. The adverse events (including signs of illness or mortality) of animals were monitored daily, and no adverse events were observed throughout the study.

After 1 week of acclimatization, the rats were assigned to one of the following six groups (*n* = 6) for 12 weeks: (1) ND (normal diet), (2) HFD (high-fat diet), (3) HFD-statin (high-fat diet, receiving lovastatin 2 mg/kg/day), (4) HFD-DR9 (high-fat diet, receiving *L. fermentum* DR9 (10 log CFU/day), (5) HFD-DR7 (high-fat diet, receiving *L. plantarum* DR7 (10 log CFU/day), (6) HFD-8513d (high-fat diet, receiving *L. reuteri* 8513d (10 log CFU/day). To induce aging, all rats were injected subcutaneously with 600 mg/kg/day of D-galactose daily. Diets used included a normal diet (ND) of standard chow diet (Altromin, Lage, Germany), a high-fat diet (HFD) containing standard chow diet augmented with 25% (*w*/*w*) animal fat (ghee, 99% fat content) (Table 1). Suspension of lactobacilli and controls were dissolved in 100 µL of saline and mixed into 1 g of food pellet prior to daily feeding. The rats were placed in individual cages during feeding of treatment to ensure complete ingestion.

### 4.3. Collection of Tissues

At the end of the twelve-week treatment period, the rats were fasted for 12 h before being sacrificed by inhalation of carbon dioxide. Blood samples were collected by cardiac puncture. All tissues were excised immediately and rinsed in saline. Tissue samples for total RNA isolation were kept in RNAlater (Sigma-Aldrich, St. Louis, MO, USA), while tissue samples for Multiplex ELISA were snap frozen and stored at −80 °C until analysis. Tissue samples for histology analysis were fixed in 10% neutral-buffered formalin.

### 4.4. Serum Biochemical Analysis

Whole blood was collected in clot activator tube, centrifuged at 1500× *g* for 15 min at 4 °C to obtain the serum, and analyzed within 48 h. Serum lipid profile (total cholesterol (TC)), TG, LDL and HDL, liver function (total protein, albumin, globulin, AG ratio), AST, ALT, ALP, total bilirubin, and renal function (sodium, urea, chloride, potassium, creatinine, uric acid, calcium, and phosphate) profile were performed in a MS ISO 15189 certified Advanced Diagnostic laboratory using an autoanalyzer AU5822 (Beckman Coulter, Brea, CA, USA) according to the method recommended by International Federation for Clinical Chemistry (IFCC).

### 4.5. Total RNA Isolation and cDNA Conversion

Total RNA was isolated using TRISURE reagent (Bioline, London, UK) and was converted to cDNA by reverse transcription using the RevertAid RT Kit (Thermo Scientific, Vilnius, Lithuania) with random hexamer primers according to manufacturer’s instructions. Briefly, 1 µg of total RNA was reverse transcribed and amplified by incubating the reaction mixture at 25 °C for 5 min, followed 42 °C for 60 min. The reaction was terminated by incubating the mixture at 70 °C for 5 min. The cDNA was diluted 10 times using nuclease-free water and used as template in qPCR or stored at −80 °C until use. The cDNA conversion was performed using the Techne Prime Thermal Cycler (Cole-Parmer, Staffordshire, UK).

### 4.6. qRT-PCR

The mRNA expression of genes involved in lipid metabolism was determined by real-time PCR analysis using the Agilent AriaMx Realtime PCR System (Agilent Technologies, Santa Clara, CA, USA). Twenty microliter PCR reactions consisted of 10 µL of 2× SensiFAST SYBR mix (Bioline, London, UK), 0.8 µL each of 10 µM forward and reverse primers, and 1 µL of cDNA. The primer sequences are shown in Table 2, using amplification conditions suggested by the manufacturer. The GAPDH gene was used as the housekeeping gene for normalization of data. The mRNA expression was expressed as fold-change relative to the control.

### 4.7. Multiplex ELISA

Frozen tissue samples were manually disrupted on a cold stainless-steel mortar and pestle and immediately homogenized in RIPA buffer (Merck, Darmstadt, Germany) containing a protease and phosphatase inhibitor cocktail (Promega, Madison, WI, USA). Supernatants were obtained by centrifugation at 10,000× *g* for 10 min at 4 °C, split into aliquots, and stored at −80 °C until analysis.

Assays were run according to the MILLIPLEX^®^ MAP Rat Cytokine/Chemokine magnetic bead panel kit (Merck, Billerica, MA, USA) instructions with overnight incubation with shaking at 4 °C (18 h, 750 rpm) and using a hand-held magnetic block for wash steps. Standards were assayed in duplicate as recommended by the manufacturer. Data were acquired on a validated and calibrated Luminex^®^ xMAP platform (Luminex, Austin, TX, USA). All data were standardized to protein concentration using the Bradford method.

### 4.8. Liver Histology

Samples fixed in neutral-buffered formalin were trimmed, followed by a dehydration and clearing step in an automatic tissue processor (Thermo Scientific^TM^ Shandon Excelsior, Kalamazoo, MI, USA). The samples were then embedded in paraffin wax and sectioned using cryotome. Sectioned samples were then stained with hematoxylin and eosin; and observed under light microscope.

### 4.9. Statistical Analysis

Data were analyzed using SPSS version 23.0 (SPSS Inc, Chicago, IL, USA). Independent *t*-tests were used to study the significant differences between sample means, with a significance level of α = 0.05. All data presented are mean values obtained from six replicates per group (*n* = 6).

## Figures and Tables

**Table 1:** Compositions of normal diet (ND) and high-fat diet (HFD) administered to rats over 12 weeks.

Content	Normal Diet (ND)		High-Fat Diet (HFD)	
	g/kg	%	g/kg	%
Crude protein	192.11	19.2	144.08	14.4
Crude fat	40.80	4.1	280.60	28.0
Crude fiber	60.74	6.1	45.56	4.6
Crude ash	58.55	5.9	43.91	4.4
Moisture	113.43	11.3	85.07	8.5
Nitrogen-free extractives	534.37	53.4	400.78	40.1
Total	1000.00	100.0	1000.00	100.0
	**kcal/kg**	**%**	**kcal/kg**	**%**
Fat	367	11.0	2525	54.0
Protein	768	24.0	576	12.3
Carbohydrates	2091	65.0	1575	33.7
Total	3226	100.0	4676	100.0

Treatments included (*n* = 6) (i) ND, (ii) HFD, (iii) HFD-statin (lovastatin 2 mg/kg/day), (iv) HFD-*L. fermentum* DR9 (10 log CFU/day), (v) HFD-*L. plantarum* DR7 (10 log CFU/day), (vi) HFD-*L. reuteri* 8513d (10 log CFU/day).

**Table 2:** List of primer sequences used for quantitative real-time polymerase chain reaction.

Target Genes	Primer Sequences (5′–3′)	NCBI Accession No.
*GAPDH*	F: CCA TCC CAG ACC CCA TAA C	NM_017008.4
	R: GCA GCG AAC TTT ATT GAT GG	
*SCD-1*	F: CAC TGG TGC CCT GGT ACT GCT	NM_139192.2
	R: GGA TGT TCT CCC GAG ATT GAA	
*IL-6*	F: TAG TCC TTC CTA CCC CAA CTT C	NM_012589.2
	R: GCC GAG TAG ACC TCA TAG TGA C	
*ABCG5*	F: TGT GAC CCT GGC ATC TAT	NM_053754.2
	R: ATC ATT GGA CCA GTT CAG T	
*ABCG8*	F: GAT GCT GGC TAT CAT AGG GAG C	NM_130414.2
	R: TCT CTG CCT GTG ATA ACG TCG A	
*SR-B1*	F: CCC AGA AGA CAC CAC GA	NM_031541.1
	R: GTG TGG ACA GTG TGA CAT CT	
*LDL-R*	F: TGG TGA CCG AGG ACA TCC AG	NM_175762.2
	R: GTG GAG TTT GGA ATC AAA CCC AAT AG	
*ABCA1*	F: CAG CAA CTA CAG TGG CGG TAA CA	NM_178095.2
	R: AAT GCT TAG GGC ACA ATT CCA CA	
*APO A1*	F: GGC AGA GAC TAT GTG TCC CAG TTT	NM_012738.1
	R: TTG AAC CCA GAG TGT CCC AGT T	
*AMPKα1*	F: GGG ATC CAT CAG CAA CTA TCG	NM_019142.2
	R: GGG AGG TCA CGG ATG AGG TA	
*AMPKα2*	F: CAT TTG TGC AAG GCC CCT AGT	NM_023991.1
	R: GAC TGT TGG TAT CTG CCT GTT TCC	

## Abbreviation

*ABCA1*	ATP-binding cassette subfamily A member 1
*ABCG5* & *ABCG8*	ATP-binding cassette subfamily G member 5 and 8
*AMPK*	5’ adenosine monophosphate-activated protein kinase
ALP	Alkaline phosphatase
ALT	Alanine aminotransferase
*Apo A1*	Apolipoprotein A1
AST	Aspartate aminotransferase
CVD	Cardiovascular disease
HDL	High-density lipoprotein
HFD	High-fat diet
*IL-6*	Interleukin-6
LDL-C	Low-density lipoprotein cholesterol
NAFLD	Non-alcoholic fatty liver disease
ND	Normal diet
*SCD1*	Stearoyl-CoA desaturase 1
*SR-B1*	Scavenger Receptor B1
TC	Total cholesterol
TG	Total triglyceride
